# The association between plant-based dietary patterns and risk of breast cancer: a case–control study

**DOI:** 10.1038/s41598-021-82659-6

**Published:** 2021-02-09

**Authors:** Somaye Rigi, Seyed Mohammad Mousavi, Sanaz Benisi-Kohansal, Leila Azadbakht, Ahmad Esmaillzadeh

**Affiliations:** 1 Department of Community Nutrition, School of Nutritional Sciences and Dietetics, Tehran University of Medical Sciences, P.O. Box 14155-6117, Tehran, Iran; 2 Students’ Scientific Research Center (SSRC), Tehran University of Medical Sciences, Tehran, Iran; 3 Diabetes Research Center, Endocrinology and Metabolism Clinical Sciences Institute, Tehran University of Medical Sciences, Tehran, Iran; 4 Obesity and Eating Habits Research Center, Endocrinology and Metabolism Molecular-Cellular Sciences Institute, Tehran University of Medical Sciences, Tehran, Iran; 5 Department of Community Nutrition, School of Nutrition and Food Science, Isfahan University of Medical Sciences, Isfahan, Iran

**Keywords:** Cancer, Diseases

## Abstract

Limited data are available, linking the plant-based diets to breast cancer (BC). We examined the association of overall plant-based diet index (PDI), hypothesized healthful (hPDI) and unhealthful versions of a plant-based diet index (uPDI) with BC in Iranian women. This population-based case–control study included 350 cases with newly diagnosed BC and 700 age-matched apparently healthy controls. We collected dietary data using a validated, Willett-format semi-quantitative food frequency questionnaire. Using these data, we generated a PDI by dedicating positive scores to plant foods, and reverse scores to animal foods, hPDI by assigning positive scores to healthy plant foods and reverse scores to less healthy plant foods and animal foods, and finally uPDI in which positive scores were assigned to less healthy plant foods and reverse scores to healthy plant foods and animal foods. After controlling for potential confounders, individuals in the highest quartile of PDI had 67% lower odds of BC than those in the lowest quartile (OR 0.33; 95% CI 0.22–0.50). Individuals with the greatest adherence to hPDI were 36% less likely to have BC than those with the lowest adherence, in the fully adjusted model (OR 0.64; 95% CI 0.43–0.94). In terms of uPDI, women in the top quartile had a 2.23 times greater chance of BC than those in the bottom quartile (OR 2.23; 95% CI 1.48–3.36). Greater adherence to PDI and hPDI was inversely associated with the risk of BC, whereas uPDI was associated with an increased risk.

## Introduction

Breast cancer (BC) ranks as the second leading cause of cancer mortality worldwide^1^. The World Health Organization reports an upward trend of 1.8–2.0% for this cancer each year across the globe^2^. According to global statistics, annually, nearly 1.4 million new cases of BC are detected, with a mortality rate of about 450,000 worldwide^3^. In Iran, based on the report of the National Cancer Registry (NCR), BC has accounted for 24.4% of various types of cancers with age-standardized rate (ASR) of 23.1 per 100,000^4^. Modifiable lifestyle factors might explain the geographic difference in BC’s incidence rate^5^, highlighting the importance of preventive approaches as promising measures to control the disease.

Heredity factors can explain less than 10% of BC etiology, while environmental, reproductive, and lifestyle factors are the main drivers of this malignancy^6^. Mounting evidence from epidemiological studies documented the effectiveness of lifestyle modifications, particularly diet on the risk of BC^7^. The majority of evidence about diet–BC associations have focused on assessing individual nutrients, food items, or food groups^8–10^. It must be noted that dietary constituents represent complex inter-relationships, and through applying defined dietary patterns, which can potentially capture the joint effect of dietary ingredients, we can rule out co-linearity drawbacks^11–13^. In this context, adherence to prudent dietary pattern^7^, Mediterranean diet^14,15^ and dietary approaches to stop hypertension eating pattern^16^ has been linked with documented benefits on BC risk. On the other hand, the Western dietary pattern^7^ has been positively associated with the risk.

Plant-based diets are a diverse family of dietary patterns defined as infrequent consumption of animal foods along with frequent intake of plant-based foods in usual diet. Vegetarian diets are a subset of plant-based diets, in which some or all animal foods have been eliminated entirely^17^. In the clinical setting, different quality of plant-based diets yielded different outcomes with respect to diet–disease relationships^18^. This highlighted the need to develop a continuous scoring system as a rigorous approach for evaluation of adherence to such dietary patterns^19^. Based on this scoring method, an overall plant-based diet index (PDI), reflecting consumption of all plant foods, was developed. In addition, a healthy plant-based diet index (hPDI), representing intake of high-quality plant foods with documented benefit on clinical outcomes, and an unhealthy plant-based diet index (uPDI), demonstrating the consumption of less nutritive plant foods with a detrimental impact on the risk of chronic conditions, were also suggested^18^. Earlier studies examining the link between these dietary patterns and the risk of chronic conditions have provided evidence indicating the predictive value of these eating habits in several conditions, including non-alcoholic fatty liver disease^20^, psychological disorder^21^, gestational diabetes mellitus^22^ and cancer^23^.

Despite several studies originating from Western nations that assessed PDI in relation to the risk of BC^24,25^, we are aware of no report in Middle-Eastern countries in this regard. This assessment in the under-studied region is of fundamental importance owing to having a traditional diet characterized by a high proportion of refined grains and detrimental fats^1^, and lower consumption of fruits, vegetables, and whole grains^26^. Considering the documented association between individual plant-based foods and breast malignancy in earlier studies^8,27–33^, it seems that the whole plant-based diet might be associated with BC. Iranian diet, which is mainly composed of plant-based foods, provides a unique opportunity to look at this association closely. Given the aforementioned points, this study was undertaken to assess the association between PDI, hPDI, and uPDI and the risk of BC in Iranian women.

## Methods and participants

### Study population

This population-based case–control study was carried out on women aged > 30 years in Isfahan, Iran, between July 2013 and July 2015. Cases were selected using a convenience sampling method from those who were referred to hospitals or private clinics. Their disease was confirmed based on physical examination, mammography, and pathological verification. Required sample size for the current work was calculated based on the hypothesis of 1.5 times increased odds of BC by unhealthy dietary pattern. Therefore, considering type I error of 5%, the study power of 80%, the common ratio of 0.25, and the ratio of controls to cases as 2, we needed 350 cases and 700 controls for this project. Patients with BC, who had a prior history of surgical resection or chemotherapy or radiotherapy or all of them, were admitted to participate in the study. BC was identified by primary incident malignant breast tumors with invasive nature in medical records. Patients with a history of any type of neoplastic lesion or cysts (except BC) and those with a prior history of any hormone replacement therapy were not included in our work. Similarly, those who were on special diets during the year before the interview were not qualified. Controls were selected randomly from healthy women. Cases and controls were matched in terms of age (± 5 years) and socioeconomic status. Inclusion criteria for control subjects were as follows: (a) being female, (b) having Iranian nationality, (c) lacking a history of any cancer, cysts, and pathological disease. The exclusion criteria for controls included adherence to special diets and having a history of hormone replacement therapy. Finally, 350 cases and 700 controls were qualified to participate in our study. Informed written consent was taken from all case and control subjects after making them aware of the study methodology. Ethical Committee of Tehran University of Medical Sciences approved the study protocol.

### Dietary intake assessment

Details on dietary intake assessment have been previously reported^16^. Briefly, to assess habitual dietary intakes (over the past year) of study participants, a 106-item Willett-format semi-quantitative dish-based food frequency questionnaire (FFQ) which was designed specifically for Iranian adults, was applied. This questionnaire included commonly-used food items and dishes in Iranian culture. Although, a classic validation study has not been conducted for this FFQ so far, several established relationships between dietary factors and several diseases using this questionnaire have been reported^34–37^. Such findings can be interpreted as qualitative support for the validity of the questionnaire^38^. A trained nutritionist conducted face-to-face interviews with participants to inquire about the average consumption frequency of foods and dishes in the FFQ. The questionnaire encompassed five sets of foods and dishes, including (1) mixed dishes (cooked or canned, 29 items); (2) carbohydrate-based foods (different sorts of bread, cakes, biscuits, and potato, 10 items); (3) milk-derived products (dairies, butter, and cream, 9 items); (4) fruits and vegetables (22 items); and (5) accessory food items and beverages (including sweets, fast foods, nuts, desserts and beverages, 36 items). Participants specified their frequency consumption for each food item and mixed dishes considering nine multiple-choice frequency response categories, ranging from “never or less than once a month” to “12 or more times per day”. In this questionnaire, for seldom-used foods, the number of multiple-choice options reduced, whereas, for frequently-used foods, more multiple-choice categories were assigned. The daily value for each item was estimated based on food composition, an average of reported frequency, and given standard size units. A trained group of dietitians used nutritionist IV software (modified for Iranian foods) to derive the average daily intake of energy and nutrients.

### Construction of plant-based dietary scores

We categorized plant foods into the healthy and less healthy groups based on epidemiological knowledge concerning the relationship between food items with several chronic conditions (type 2 diabetes, cardiovascular disease, and certain cancers) along with intermediate conditions (obesity, hypertension or inflammation)^18^. We generated 16 food groups (belonged to the whole collection of animal foods, healthy and less healthy plant foods) according to nutrient and culinary standard features. After accounting for daily values for each food item, the number of servings for entire foods incorporated in each of the 16 food groups were summed up. In our project, we generated an overall plant-based diet index (PDI) according to the algorithm developed by Martinez-Gonzalez et al.^19^ and two fitted versions of a healthful plant-based diet index (hPDI), and an unhealthful plant-based diet index (uPDI), as suggested by Satija et al.^18^. Whole grains, fruits, vegetables, nuts, legumes, and vegetable oils were classified in the category of healthy plant food groups; fruit juices, sugar-sweetened beverages, refined grains, and potatoes were located in less healthy plant food groups; animal food groups encompassed the wide range of animal fats, dairy, eggs, fish/seafood, meat (poultry and red meat) and miscellaneous animal-based foods. The cut points for the quintiles were calculated for each 16 food groups with assigned score between 1 and 5 for each quintile. Regarding PDI, the score of 5 was dedicated for each plant food groups which were above the highest quintile of consumption, a score of 4 was assigned to each plant food groups which were above the second-highest quintile and below the highest quintile, and so on, at last participants received a score of 1 for each plant food groups for which they were below the lowest quintile of consumption (positive scores). In contrast, a score of 1 was allocated to each animal food groups upper the highest quintile of consumption, a score of 2 was dedicated to each animal food groups incorporated between the highest and second highest quintiles, and so on, at last a score of 5 for consumption under the bottom quintile (reverse scores). To calculate hPDI, scores of 5 and 1 were given to subjects with the highest and lowest consumption of healthy plant foods, respectively. A score of 1 for the highest consumption and 5 for the lowest consumption of unhealthy plant foods and animal food items was also given. To calculate uPDI, a score between 5 and 1 was given to the highest through the lowest consumption of unhealthy plant foods. Furthermore, subjects with the highest to lowest consumption of animal foods and healthy plant foods were given a score between 1 and 5. To achieve each participant’s indices, we added up 16 food group scores. Though we theoretically reached the range of 16 (as the lowest possible score) to 80 (as the highest possible score) for these indices, in practice the observed score ranges for PDI, hPDI, and uPDI, were 21–74, 26–77, and 31–78, respectively, across the groups. It is worth mentioning that a higher amount of all indices is indicative of a lower intake of animal foods. Alcoholic beverages were not included in our indices due to their diverse association with several health outcomes.

### Assessment of breast cancer

Diagnosis of BC was made by physical examination and mammography; pathological assessments determined final confirmation of BC. Only females with newly diagnosed (maximum 6 months since diagnosis) BC (stage I–IV) with Iranian nationality were recruited into this study.

### Assessment of covariates

A general information pretested questionnaire including several variables of sociodemographic status (age, marital status, residential place, and education), alcohol consumption, smoking, menopausal status, disease history, family history of BC, history of breastfeeding and supplement use was administered to collect general information of subjects. A short form of the International Physical Activity Questionnaire (IPAQ) was applied through face-to-face interviews. The acquired information from the IPAQ were stated as Metabolic Equivalent-hours per week (METs/week). Anthropometric measures were assessed, and participants’ BMI was calculated accordingly.

### Statistical analysis

We classified all subjects based on the PDI, hPDI and uPDI scores into quartile ranges. The Chi-square test was applied to assess the distribution of study participants in terms of general characteristics between cases and controls. To compare the means of continuous variables, including dietary intakes between cases and controls, one-way analysis of variance (ANOVA) was used. Comparisons across quartiles of PDI, hPDI, and uPDI were performed using one-way ANOVA. We used multivariable logistic regression to investigate the association of PDI, hPDI, and uPDI with BC. We reached the ORs and 95% CIs using three different models: i.e., Model 1: Adjusted for age and energy intake, Model 2: Adjusted for education, social-economic status, residential area, supplement use, family history of BC, disease history, physical activity, marital status, smoking status, alcohol consumption, history of breast-feeding and menopausal status, Model 3: Adjusted for body mass index(BMI). Selection of covariates was done according to the previous studies on BC that have introduced contributing factors to this condition^39–44^. All the statistical analyses were performed using SPSS (SPSS Inc., version 19). Significant results were characterized, considering two-sided p < 0.05.

### Ethical approval

All procedures performed in our study that involved human participants were in accordance with the ethical standards of the institutional and/or national research committee and with the 1964 Helsinki declaration and its later amendments or comparable ethical standards.

### Informed consent

Informed consent was obtained from all individual participants included in the study.

## Results

Overall, 1050 participants with a mean age of 62.4 years were included. Baseline characteristics and dietary intakes of study participants separately by cases and controls are provided in Table 1. Women with BC were more likely to be older, post menopause, and have a family history of BC than control subjects. Moreover, they had lower BMI and were less likely to be married and have higher educational levels than those without BC. In terms of dietary intakes, they were more likely to have higher intakes of total energy, carbohydrate, total fat, cholesterol, SFA, MUFA, vitamin B6, calcium, magnesium, potassium, fruits, sugar-sweetened beverages, sweet dessert, animal fats, tea and coffee as well as lower intakes of PUFA, whole grains, vegetables, nuts, legumes, vegetable oils, fruit juices, potato, and meats compared with controls.

**Table 1:** Demographic characteristics and dietary intakes of cases and controls.

	Groups		
	Controls (*n* = 700)	Cases (*n* = 350)	P*
Age (years)	61.4 ± 10.3	65.2 ± 11.2	< 0.001
BMI (kg/m^2^)	25.5 ± 5	21.8 ± 4.8	< 0.001
Married (%)	88.3	74.6	< 0.001
Educated (%)	28.9	17.4	< 0.001
Low SES (%)	29	33.4	0.31
Urban-resided (%)	36.1	36	0.96
Family history of breast cancer (%)	3.4	9.4	< 0.001
History of breast feeding (%)	33.7	34.0	0.92
Disease history (%)	8.7	10.3	0.40
Current smoker (%)	13	17.4	0.05
Alcohol consumption (%)	7.4	4.6	0.07
Supplement use (%)	10.1	9.4	0.71
Post menopause (%)	77.4	88.3	< 0.001
Physical activity (MET/h)	34.8 ± 6.5	35.4 ± 6.7	0.19
**Nutrient items**			
Total energy (kcal/day)	2177 ± 608	2499 ± 793	< 0.001
Carbohydrate (g/day)	305 ± 96	340 ± 123	< 0.001
Protein (g/day)	77.0 ± 27.7	78.6 ± 30.3	0.38
Total fat (g/day)	77.5 ± 28.3	98.6 ± 42.0	< 0.001
Dietary fiber (g/day)	22.2 ± 7.6	22.7 ± 8.4	0.33
Cholesterol (mg/day)	182 ± 97.3	204.7 ± 134	0.002
SFA (g/day)	26.1 ± 19.9	44.1 ± 33.2	< 0.001
PUFA (g/day)	10.5 ± 8.1	9.0 ± 4.4	< 0.001
MUFA (g/day)	19.8 ± 7.7	21.3 ± 9.3	0.01
Thiamine (mg/day)	1.95 ± 0.65	2.03 ± 0.66	0.07
Vitamin B6 (mg/day)	1.58 ± 0.49	1.65 ± 0.59	0.04
Folate (µg/day)	276 ± 88	287 ± 123	0.11
Vitamin B12 (µg/day)	2.65 ± 2.1	2.98 ± 2.8	0.06
Calcium (mg/day)	736 ± 276	821 ± 330	< 0.001
Magnesium (mg/day)	442 ± 141	472 ± 156	0.002
Potassium (mg/day)	2850 ± 817	3070 ± 1128	< 0.001
**Food groups (g/day)**			
Whole grains	325 ± 129	299 ± 130	0.002
Fruits	150 ± 115	201 ± 172	< 0.001
Vegetables	145 ± 92	111 ± 76	< 0.001
Nuts	3.1 ± 6.1	0.74 ± 3.9	< 0.001
Legumes	15.9 ± 15.4	12.4 ± 12.6	< 0.001
vegetable oils	2.6 ± 7.8	0.58 ± 2.5	< 0.001
Tea and coffee	1319 ± 756	1486 ± 1290	0.02
Refined grains	97.1 ± 68.9	90.1 ± 79.0	0.16
Sugar-sweetened beverages	34.1 ± 33.4	64.2 ± 90.6	< 0.001
Fruit juices	1.8 ± 3.8	1.0 ± 3.1	< 0.001
Potato	63.6 ± 41.7	55.8 ± 61.5	0.03
Sweet dessert	52.5 ± 34.9	63.7 ± 64	< 0.001
Animal fats	30.4 ± 19.7	42.6 ± 29.0	< 0.001
Dairy	228 ± 135	241 ± 161	0.20
Eggs	11.0 ± 12.1	11.4 ± 18.3	0.69
Fish/seafood	8.4 ± 13.1	10.9 ± 50.3	0.36
Meats	83.6 ± 64.7	68.8 ± 69.2	0.001

All values are mean ± SD or percent.

*BMI* body mass index, *MET* metabolic equivalents, *SES* social economic status, *SFA* saturated fatty acid, *PUFA* polyunsaturated fatty acid, *MUFA* monounsaturated fatty acid.

*Obtained from independent sample *t* test or Chi-square test, where appropriate.

General characteristics and dietary intakes of participants according to quartile categories of PDI, hPDI and uPDI are described in Tables 2 and 3, respectively. Higher PDI score was associated with greater BMI, higher educational level, and younger age. Also, a lower percentage of subjects in the highest category of PDI had disease history than those in the lowest category. In addition, the highest PDI was accompanied by higher intakes of fruits, vegetables, nuts, legumes, vegetable oils, tea and coffee, refined grains, sugar-sweetened beverages, fruit juices, potato, sweet dessert, and a lower intake of total energy, carbohydrate, protein, total fat, cholesterol, SFA, MUFA, thiamine, vitamin B6, vitamin B12, calcium, magnesium, potassium, animal fats, dairy, eggs, fish/seafood, and meat. Compared with subjects in the lowest quartile, women in the top quartile of hPDI were more likely to be alcohol users and less likely to be older. They also had higher intakes of carbohydrates, dietary fiber, PUFA, thiamine, vitamin B6, folate, magnesium, potassium, whole grains, fruits, vegetables, nuts, legumes, vegetable oils, tea, and coffee and lower intakes of total energy, total fat, cholesterol, SFA, MUFA, vitamin B12, calcium, refined grains, sugar-sweetened beverages, potato, animal fats, dairy, eggs, fish/seafood, and meats. In terms of uPDI, a greater percentage of subjects in the highest category of uPDI were married and had low socioeconomic status than those in the lowest category. Higher uPDI was associated with lower BMI. Individuals in the highest category of uPDI were less likely to be university graduated, alcohol users, and live in urban areas than those in the lowest category. Moreover, they consumed higher amounts of total energy, carbohydrates, proteins, total fat, dietary fiber, SFA, MUFA, thiamine, vitamin B6, folate, calcium, magnesium, potassium, whole grains, tea and coffee, sugar-sweetened beverages, potato, sweet dessert and lower amounts of fruits, vegetables, nuts, legumes, vegetable oils, fruit juices, dairy, eggs, fish/seafood and meats than those with the lowest adherence to this index.

**Table 2:** Demographic characteristics of study participants across quartiles of PDI, hPDI, and uPDI scores.

	Quartiles of PDI					Quartiles of hPDI					Quartiles of uPDI				
	Q1	Q2	Q3	Q4	P*	Q1	Q2	Q3	Q4	P*	Q1	Q2	Q3	Q4	P*
Participants (n)	251	282	237	280		276	224	272	278		246	273	247	284	
Age (years)	64.2 ± 10.7	62.7 ± 11.4	61.7 ± 10.7	61.2 ± 10.2	0.01	63.8 ± 10	62.4 ± 10.2	61.8 ± 10.7	61.6 ± 10.4	0.04	61.9 ± 11.2	62.3 ± 10.7	63.5 ± 10.8	62.1 ± 10.5	0.33
BMI (kg/m2)	23.6 ± 5.8	24.1 ± 5.3	25 ± 5	24.5 ± 4.8	0.01	23.8 ± 5.4	24.3 ± 5.2	24.8 ± 5.7	24.3 ± 4.7	0.16	25.1 ± 5.3	24.8 ± 5.2	23.9 ± 5.2	23.4 ± 5.2	0.001
Married (%)	78.5	83.7	85.2	87.1	0.11	81.5	83	82.7	87.4	0.32	87.4	78	78.9	90.1	0.001
Educated (%)	17.5	28.7	27.4	26.1	0.01	22.1	26.8	28.3	23.4	0.30	39.8	23.8	21.9	16.2	< 0.001
Low SES (%)	36.3	28	27.4	30.4	0.09	26.8	30.4	33.5	31.3	0.53	20.3	26.4	32.4	41.5	< 0.001
Urban-resided (%)	32.3	35.5	36.3	40	0.32	33.3	40.2	39.3	32.4	0.14	53.3	37	33.6	22.5	< 0.001
Family history of breast cancer (%)	6.8	3.9	3.8	7.1	0.17	5.1	6.7	6.6	3.6	0.34	3.7	7	4	6.7	0.20
History of breast feeding (%)	33.1	36.9	33.3	31.8	0.62	36.2	33.9	34.2	30.9	0.62	38.2	31.9	31.2	34.2	0.34
Disease history (%)	13.5	7.8	8.9	6.8	0.02	11.2	7.1	9.2	9	0.47	9.3	12.5	7.3	7.7	0.15
Current smoker (%)	17.9	12.8	12.2	15	0.25	13.4	14.7	16.5	13.3	0.68	10.6	15.8	13.8	17.3	0.15
Alcohol consumption (%)	4.8	6.7	5.9	8.2	0.43	4.7	4.9	10.3	5.8	0.03	10.6	7.7	4.5	3.5	0.004
Supplement use (%)	6.8	11	10.5	11.1	0.29	9.1	10.7	9.9	10.1	0.94	6.9	9.2	10.5	12.7	0.16
Post menopause (%)	86.1	78	79.7	80.7	0.11	84.8	77.7	80.9	80.2	0.23	80.5	81	82.2	80.6	0.96
Physical activity (MET/h)	35 ± 6.7	35.5 ± 6.7	34.6 ± 6.6	35 ± 6.4	0.52	35.2 ± 7	34.6 ± 5.8	35.6 ± 6.9	34.6 ± 6.4	0.30	35.3 ± 6.4	35 ± 6.8	34.9 ± 6.5	34.8 ± 6.7	0.86

All values are mean ± SD or percent.

*BMI* body mass index, *MET* metabolic equivalents, *SES* social economic status, *PDI* overall plant-based diet index, *hPDI* healthy plant-based diet index, *uPDI* unhealthy plant-based diet index.

*Obtained from ANOVA or Chi-square test, where appropriate.

**Table 3:** Dietary intakes of study participants across quartiles of PDI, hPDI, and uPDI scores.

	Quartiles of PDI					Quartiles of hPDI					Quartiles of uPDI				
	Q1	Q2	Q3	Q4	P*	Q1	Q2	Q3	Q4	P*	Q1	Q2	Q3	Q4	P*
**Nutrients**															
Total energy (kcal/day)	2654 ± 725	2279 ± 602	2095 ± 662	2119 ± 641	< 0.001	2354 ± 768	5157 ± 686	2251 ± 665	2351 ± 692	0.004	2055 ± 665	2027 ± 631	2285 ± 631	2731 ± 594	< 0.001
Carbohydrate (g/day)	334 ± 119	311 ± 99	299 ± 100	323 ± 106	0.002	304 ± 115	291 ± 103	313 ± 93	654 ± 106	< 0.001	276 ± 95	268 ± 81	325 ± 99	392 ± 101	< 0.001
Protein (g/day)	92 ± 35	78 ± 23	73 ± 27	66 ± 21	< 0.001	80 ± 31	74 ± 29	76 ± 28	77 ± 24	0.11	74 ± 24	73 ± 27	76 ± 29	84 ± 31	< 0.001
Total fat (g/day)	111 ± 42	86 ± 25	73 ± 29	68 ± 24	< 0.001	96 ± 36	82 ± 35	83 ± 36	76 ± 28	< 0.001	78 ± 31	78 ± 36	81 ± 29	98 ± 36	< 0.001
Dietary fiber (g/day)	22.5 ± 8.4	22.4 ± 7.4	21.4 ± 7.8	23.2 ± 7.8	0.08	19.5 ± 7.1	19.9 ± 6.9	22.7 ± 7.2	27 ± 7.8	< 0.001	21.7 ± 8.3	19.6 ± 6.7	22.4 ± 7.5	25.7 ± 7.6	< 0.001
Cholesterol (mg/day)	253 ± 135	198 ± 101	172 ± 95	138 ± 75	< 0.001	237 ± 125	190 ± 96	184 ± 109	146 ± 89	< 0.001	205 ± 96	191 ± 118	180 ± 106	181 ± 119	0.04
SFA (g/day)	50.2 ± 38.4	32.6 ± 20.1	25.4 ± 16.9	20.9 ± 14.8	< 0.001	39.8 ± 26.1	34.6 ± 32.4	30.8 ± 26.6	23.5 ± 17.5	< 0.001	25.9 ± 22.9	29 ± 23.8	30.5 ± 20.5	41.7 ± 33.3	< 0.001
PUFA (g/day)	10.1 ± 4.8	9.8 ± 5.2	9.7 ± 10.5	10.3 ± 6.7	0.72	9.6 ± 4.5	8.5 ± 4.1	10 ± 6.2	11.6 ± 10.7	< 0.001	11 ± 8.1	9.4 ± 9.6	9.5 ± 5.1	10.1 ± 4.0	0.07
MUFA (g/day)	24 ± 9.3	21 ± 7.2	18.2 ± 7.8	18 ± 7.3	< 0.001	21.5 ± 8.4	18.8 ± 7.5	20.1 ± 8.6	20.4 ± 8.2	0.003	18.8 ± 7.5	18.5 ± 8.9	20.3 ± 7.8	23.2 ± 7.9	< 0.001
Thiamine (mg/day)	2.1 ± 0.7	2.0 ± 0.6	1.8 ± 0.6	1.9 ± 0.6	< 0.001	1.8 ± 0.6	1.8 ± 0.6	2.0 ± 0.6	2.3 ± 0.6	< 0.001	1.8 ± 0.6	1.7 ± 0.5	2.0 ± 0.6	2.4 ± 0.6	< 0.001
Vitamin B6 (mg/day)	1.8 ± 0.6	1.6 ± 0.5	1.5 ± 0.5	1.5 ± 0.5	< 0.001	1.6 ± 0.5	1.5 ± 0.5	1.6 ± 0.5	1.7 ± 0.5	< 0.001	1.6 ± 0.5	1.5 ± 0.5	1.6 ± 0.5	1.8 ± 0.5	< 0.001
Folate (µg/day)	281 ± 114	278 ± 96	267 ± 96	289 ± 95	0.10	265 ± 102	255 ± 98	280 ± 95	313 ± 92	< 0.001	281 ± 103	250 ± 94	281 ± 106	305 ± 92	< 0.001
Vitamin B12 (µg /day)	3.4 ± 2.8	2.8 ± 2.2	2.4 ± 1.9	2.3 ± 2.5	< 0.001	3.3 ± 2.5	2.7 ± 2	2.8 ± 3	2.2 ± 1.6	< 0.001	2.9 ± 1.7	2.7 ± 2.6	2.6 ± 2.7	2.8 ± 2.4	0.73
Calcium (mg/day)	879 ± 303	787 ± 311	690 ± 250	702 ± 283	< 0.001	805 ± 316	726 ± 308	754 ± 283	766 ± 281	0.02	783 ± 290	732 ± 300	733 ± 274	808 ± 316	0.006
Magnesium (mg/day)	489 ± 160	449 ± 139	430 ± 141	438 ± 139	< 0.001	413 ± 142	405 ± 140	451 ± 131	527 ± 141	< 0.001	423 ± 143	395 ± 125	455 ± 140	528 ± 142	< 0.001
Potassium (mg/day)	3080 ± 1054	2888 ± 868	2766 ± 890	2951 ± 914	0.004	2883 ± 954	2700 ± 943	2873 ± 833	3192 ± 956	< 0.001	2923 ± 1001	2599 ± 833	2906 ± 971	3249 ± 834	< 0.001
**Food groups (g/day)**															
Whole grains	302 ± 154	319 ± 124	331 ± 117	315 ± 120	0.12	248 ± 106	290 ± 109	321 ± 114	401 ± 133	< 0.001	302 ± 107	296 ± 124	327 ± 120	339 ± 154	< 0.001
Fruits	147 ± 172	147 ± 112	174 ± 123	199 ± 136	< 0.001	146 ± 120	169 ± 139	175 ± 142	178 ± 150	0.02	233 ± 134	173 ± 118	157 ± 126	113 ± 147	< 0.001
Vegetables	87 ± 60	132 ± 91	141 ± 82	170 ± 93	< 0.001	110 ± 66	129 ± 72	135 ± 85	158 ± 112	< 0.001	189 ± 116	139 ± 70	121 ± 61	90 ± 67	< 0.001
Nuts	0.4 ± 3.3	2.5 ± 7.3	2.6 ± 4.4	3.6 ± 5.6	< 0.001	1.5 ± 4.4	1.8 ± 2.9	2.8 ± 7	3.2 ± 6.4	0.001	3.6 ± 5.2	2.6 ± 4.6	2.1 ± 5.4	1.2 ± 6.5	< 0.001
Legumes	8.3 ± 10.3	14.9 ± 12.8	16.3 ± 20.4	19.1 ± 12.1	< 0.001	12.1 ± 12.2	13.6 ± 11.6	14.6 ± 10.6	18.5 ± 20.6	< 0.001	18.4 ± 11	17.3 ± 20.5	14.1 ± 11.2	9.7 ± 11.5	< 0.001
Vegetable oils	0.2 ± 3.0	1.3 ± 5.9	2.1 ± 7.3	3.9 ± 8.3	< 0.001	0.6 ± 3.9	0.6 ± 2.4	2.1 ± 6.6	4.1 ± 9.7	< 0.001	4.8 ± 10.7	1.8 ± 5.4	1.3 ± 5.1	0.09 ± 1	< 0.001
Tea and coffee	1170 ± 1190	1258 ± 824	1423 ± 752	1636 ± 992	< 0.001	1252 ± 877	1338 ± 983	1314 ± 812	1586 ± 1145	< 0.001	1377 ± 708	1221 ± 728	1496 ± 1099	1417 ± 1207	0.003
Refined grains	82 ± 73	89 ± 79	97 ± 54	109 ± 75	< 0.001	112 ± 70	96 ± 69	100 ± 82	70 ± 58	< 0.001	91 ± 71	98 ± 74	93 ± 72	96 ± 72	0.75
Sugar-sweetened beverages	38.6 ± 74.3	38 ± 48.8	48.7 ± 70.6	51.4 ± 46	0.004	54.8 ± 61.9	60.4 ± 83.3	35.8 ± 50.3	28.6 ± 37.6	< 0.001	32.8 ± 32.6	46 ± 58.6	45.5 ± 60.1	51 ± 78	< 0.001
Fruit juices	0.6 ± 2.3	1.4 ± 3.8	1.6 ± 3	2.5 ± 4.4	< 0.001	1.3 ± 2.6	1.8 ± 3.9	1.8 ± 4.5	1.3 ± 3.2	0.26	2.7 ± 4.9	1.8 ± 4.1	1.0 ± 2.1	1.1 ± 2.4	< 0.001
Potato	48.7 ± 57.5	65.8 ± 48.5	58.3 ± 35.4	69.7 ± 50.2	< 0.001	71.7 ± 49.9	58.5 ± 42.5	56.1 ± 46.1	57.3 ± 55.2	0.001	42.4 ± 31.7	53.8 ± 38.4	68 ± 50.2	78.2 ± 62.1	< 0.001
Sweet dessert	48.5 ± 63.5	50.8 ± 34.2	56.5 ± 35.1	68.5 ± 47.1	< 0.001	60.6 ± 47.9	59 ± 37.8	54.3 ± 43.5	51.6 ± 54.9	0.09	44.4 ± 27.4	51.6 ± 29.3	59.5 ± 53.1	68.2 ± 62.9	< 0.001
Animal fats	46.1 ± 35.9	35.4 ± 19.1	31 ± 15.3	26.1 ± 14.9	< 0.001	41.4 ± 22.6	39 ± 29.1	34.3 ± 22.4	24.2 ± 17.6	< 0.001	33.4 ± 20.5	36.2 ± 21.6	32.7 ± 20.1	35.4 ± 30.8	0.22
Dairy	265 ± 160	248 ± 163	214 ± 124	203 ± 115	< 0.001	283 ± 153	246 ± 157	219 ± 129	183 ± 119	< 0.001	274 ± 123	254 ± 127	211 ± 127	194 ± 174	< 0.001
Eggs	13.4 ± 20.7	11.5 ± 13.4	11.2 ± 11.7	8.6 ± 9.8	0.001	15.5 ± 17.3	11.3 ± 13.6	10.4 ± 13.9	7.3 ± 11.1	< 0.001	15.6 ± 14.5	12.7 ± 13.8	10.2 ± 14.3	6.5 ± 13.7	< 0.001
Fish/seafood	13.7 ± 58.9	7.8 ± 12.5	9.9 ± 16.3	6.0 ± 8.5	0.002	16.3 ± 56.1	8.4 ± 12.1	7.9 ± 13.3	4.2 ± 11.3	< 0.001	15.5 ± 16.1	11 ± 23.3	4.5 ± 8.4	6.1 ± 51.6	< 0.001
Meats	97.7 ± 97.4	81.6 ± 56.3	80.8 ± 57.6	57.3037.1 ±	< 0.001	87.1 ± 68.2	88.1 ± 62.7	77 ± 75.5	62.5 ± 55.1	< 0.001	81.4 ± 40.8	88.7 ± 57.8	80.2 ± 67.3	65.4 ± 87	0.003

All values are mean ± SD.

*BMI* body mass index, *MET* metabolic equivalents, *SES* social economic status, *PDI* overall plant-based diet index, *hPDI* healthy plant-based diet index, *uPDI* unhealthy plant-based diet index.

*Obtained from ANOVA.

Table 4 outlines the crude and multivariable-adjusted ORs and 95% CIs for BC across quartiles of PDI, hPDI and uPDI. In the crude model, women in the top quartile of PDI had 77% lower odds of BC than those in the bottom quartile (OR 0.23; 95% CI 0.15–0.33). After adjustment for age, energy intake, and several demographic confounders, this association remained significant but slightly attenuated (OR 0.33; 95% CI 0.22–0.49). When BMI was taken into account, this association did not alter (OR 0.33; 95% CI 0.22–0.50). Such findings were also seen with regard to hPDI; such that there was a similar decreasing trend of odds ratios across increasing quartiles of hPDI, either before or after adjustment for confounders. For instance, after controlling for potential confounders, including BMI, individuals with the greatest adherence to hPDI were 36% less likely to have BC than those with the lowest adherence (OR 0.64; 95% CI 0.43–0.94). In terms of uPDI, we found evidence for increased odds for BC across increasing quartiles. Women in the top quartile of this index had a 2.12 times greater chance of BC than those in the bottom quartile (OR 2.12; 95% CI 1.46–3.08). The finding remained unchanged in the fully adjusted model (OR 2.23; 95% CI 1.48–3.36).

**Table 4:** Crude and multivariable-adjusted ORs and 95% CIs for breast cancer across quartiles of PDI, hPDI, and uPDI scores.

	PDI					hPDI					uPDI				
	Q1	Q2	Q3	Q4	P_trend_	Q1	Q2	Q3	Q4	P_trend_	Q1	Q2	Q3	Q4	P_trend_
**Breast cancer**															
Crude	1.00	0.29 (0.20–0.42)	0.18 (0.12–0.27)	0.23 (0.15–0.33)	< 0.001	1.00	0.81 (0.56–1.16)	0.65 (0.46–0.93)	0.57 (0.40–0.81)	0.001	1.00	1.27 (0.86–1.88)	1.77 (1.20–2.61)	2.12 (1.46–3.08)	< 0.001
Model I	1.00	0.35 (0.24–0.52)	0.25 (0.16–0.38)	0.31 (0.21–0.46)	< 0.001	1.00	1.00 (0.68–1.47)	0.75 (0.59–1.09)	0.61 (0.42–0.89)	0.005	1.00	1.27 (0.85–1.89)	1.70 (1.14–2.52)	2.17 (1.48–3.17)	< 0.001
Model II	1.00	0.39 (0.26–0.58)	0.27 (0.17–0.42)	0.33 (0.22–0.49)	< 0.001	1.00	1.04 (0.69–1.56)	0.73 (0.50–1.08)	0.64 (0.43–0.94)	0.008	1.00	1.09 (0.72–1.65)	1.64 (1.08–2.48)	2.23 (1.48–3.35)	< 0.001
Model III	1.00	0.39 (0.26–0.58)	0.27 (0.17–0.42)	0.33 (0.22–0.50)	< 0.001	1.00	1.04 (0.69–1.56)	0.74 (0.50–1.09)	0.64 (0.43–0.94)	0.008	1.00	1.09 (0.72–1.65)	1.64 (1.08–2.48)	2.23 (1.48–3.36)	< 0.001

Data are presented as OR (95% CI).

Model I: Adjusted for age and energy intake.

Model II: Additionally, adjusted for education, social economic status, residential area, supplement use, family history of breast cancer, disease history, physical activity, marital status, smoking status, alcohol consumption, history of breast-feeding and menopausal status.

Model III: Further adjustment for BMI.

When we made stratified analysis by menopausal status, PDI was inversely associated with BC risk in both pre- (OR 0.23; 95% CI 0.59–0.94) and post-menopausal women (OR 0.35; 95% CI 0.22–0.56) (Table 5). There was an inverse association between hPDI and odds of postmenopausal BC (OR for the highest vs. lowest quartile 0.62; 95% CI 0.41–0.95). No significant association was found between hPDI and odds of BC among premenopausal women. A higher score of uPDI was positively associated with risk of postmenopausal BC (OR 2.42; 95% CI 1.51–3.87). No significant association was found between uPDI and odds of BC among premenopausal women.

**Table 5:** Crude and multivariable-adjusted ORs and 95% CIs for breast cancer across quartiles of PDI, hPDI, and uPDI scores stratified based on menopausal status.

	PDI					hPDI					uPDI				
	Q1	Q2	Q3	Q4	P_trend_	Q1	Q2	Q3	Q4	P_trend_	Q1	Q2	Q3	Q4	P_trend_
**Premenopausal**															
Crude	1.00	0.25 (0.09–0.66)	0.31 (0.11–0.82)	0.20 (0.07–0.56)	0.006	1.00	4.16 (1.38–12.4)	1.55 (0.48–5.03)	1.45 (0.45–4.69)	0.66	1.00	0.52 (0.18–1.48)	0.86 (0.32–2.34)	1.15 (0.46–2.84)	0.52
Model I	1.00	0.31 (0.12–0.84)	0.39 (0.14–1.09)	0.28 (0.09–0.83)	0.04	1.00	3.61 (1.17–11.1)	1.36 (0.40–4.53)	1.15 (0.34–3.86)	0.39	1.00	0.55 (0.19–1.57)	0.94 (0.34–2.60)	1.24 (0.49–3.12)	0.29
Model II	1.00	0.31 (0.10–0.92)	0.47 (0.15–1.48)	0.26 (0.08–0.88)	0.08	1.00	3.47 (1.01–11.8)	1.38 (0.39–4.91)	1.00 (0.28–3.56)	0.34	1.00	0.49 (0.15–1.59)	0.70 (0.23–2.10)	1.13 (0.38–3.28)	0.65
Model III	1.00	0.21 (0.05–0.85)	0.72 (0.16–3.16)	0.23 (0.59–0.94)	0.19	1.00	3.03 (0.71–12.8)	0.59 (0.13–2.74)	0.54 (0.11–2.64)	0.23	1.00	0.78 (0.20–3.03)	1.61 (0.42–6.23)	1.28 (0.36–4.52)	0.51
**Postmenopausal**															
Crude	1.00	0.31 (0.21–0.47)	0.17 (0.11–0.26)	0.24 (0.16–0.35)	< 0.001	1.00	0.65 (0.43–0.97)	0.61 (0.42–0.89)	0.53 (0.36–0.78)	0.001	1.00	1.49 (0.97–2.28)	2.00 (1.31–3.07)	2.42 (1.60–3.65)	< 0.001
Model I	1.00	0.36 (0.24–0.55)	0.23 (0.14–0.36)	0.32 (0.21–0.49)	< 0.001	1.00	0.79 (0.52–1.22)	0.72 (0.48–1.07)	0.58 (0.39–0.87)	0.008	1.00	1.50 (0.97–2.30)	1.94 (2.26–2.99)	2.49 (1.64–3.78)	< 0.001
Model II	1.00	0.40 (0.26–0.62)	0.25 (0.15–0.41)	0.34 (0.22–0.54)	< 0.001	1.00	0.84 (0.54–1.32)	0.71 (0.47–1.08)	0.62 (0.41–0.93)	0.02	1.00	1.28 (0.81–2.01)	1.89 (1.20–2.98)	2.65 (1.67–4.11)	< 0.001
Model III	1.00	0.37 (0.24–0.59)	0.26 (0.16–0.43)	0.35 (0.22–0.56)	< 0.001	1.00	1.03 (0.64–1.65)	0.93 (0.59–1.47)	0.62 (0.41–0.95)	0.04	1.00	1.21 (0.75–1.93)	1.64 (1.02–2.64)	2.42 (1.51–3.87)	< 0.001

Data are presented as OR (95% CI).

Model I: Adjusted for age and energy intake.

Model II: Additionally, adjusted for education, social economic status, residential area, supplement use, family history of breast cancer, disease history, physical activity, marital status, smoking status, alcohol consumption, history of breast-feeding.

Model III: Further adjustment for BMI.

## Discussion

In the current population-based case–control study, we found a significant inverse association between PDI and hPDI scores and the likelihood of having BC, whereas there was a significant direct association between higher uPDI score and odds of BC. These associations remained significant even after considering potential confounders. Such associations were also seen in postmenopausal women. Though PDI was inversely associated with BC risk in premenopausal women, we failed to find any significant association between hPDI, uPDI and odds of premenopausal BC. To the best of our knowledge, this study is the first investigation that examined the association between adherence to plan-based dietary indices and odds of BC in the Middle East.

Carcinoma of the breast as a major health concern represents a progressively increasing trend and is subject to decreased age at the onset, making it a major cost of the healthcare system in the future^45,46^. Thus, effective primary preventions have important implications for relieving the rising burden of BC. Epidemiological evidence support the role of diet in BC's pathogenesis, so that it may facilitate or increase the risk of BC^47–49^. The results of the current study suggested an inverse association between higher adherence to PDI and hPDI scores and odds of BC, while the uPDI score was positively related to the chance of BC. Such associations were also seen in postmenopausal women. Though PDI was inversely associated with BC risk in premenopausal women we failed to find any significant association between hPDI, uPDI and odds of premenopausal BC. Published studies on plant-based dietary indices and BC have found little evidence of an association, which is inconsistent with our results. In the French cohort study of 487 BC survivors, though the greater plant-based dietary score was related to decreased overall cancer recurrence, the tumor-specific assessment showed a non-significant association with BC incidence^25^. In contrary, Martinez-Gonzalez and colleagues found that pro-plant-based score was inversely associated with all-cause mortality among omnivorous participants, nevertheless stratified analysis revealed non-significant association for cancer deaths^50^. Notably, a small number of cancer deaths could undermine statistical power to detect any small association in this regard. In another cohort study of 10,812 Spanish women, the investigators reported inverse associations between moderate adherence to overall pro-vegetarian dietary patterns (PVG) and BC risk, but the healthful PVG (hPVG) and unhealthful PVG (uPVG) had no association with BC risk^51^. Our findings were comparable with those from several investigations evaluating the association between BC risk and: (1) overall diet, encompasses plant-based and animal components, determined by a priori (dietary patterns) or posteriori (dietary scores) procedures, or (2) vegetarian and/or vegan diets. In line with our findings, inverse associations were seen between dietary scores assessing compliance to numerous healthy dietary patterns including the Mediterranean Diet Score (MDS)^14^, the Healthy Eating Index (HEI)^52^ and the Dietary Approaches to Stop Hypertension score (DASH)^53^ in relation to the risk of BC. In a recent meta-analysis, individuals with greater adherence to Western dietary pattern were 14% more likely to have BC compared with those with the lowest adherence, while consumption of a prudent dietary pattern was associated with an 18% reduced risk of BC^7^. In a recent systematic review on dietary patterns and risk of BC, lower risk of BC was observed with a healthy dietary pattern comprising of vegetables as well as fruits, legumes and whole grains while higher risk was observed with a dietary pattern high in saturated fats and red and processed meats as well as added sugars, fried foods and refined grains^54^. Similarly, according to summary results of a recent publication, a higher dietary colored non-starchy vegetable intake was associated with a reduced risk for BC, while high intakes of red and processed meats, high energy dense and high glycemic foods and beverages were positively associated with the risk of BC^55^. For vegetarian diets existing data are limited, but a meta-analysis of cohort studies reported no significant association between exclusive vegetarian diet and BC^56^. In line with our results, Dinu et al., in a meta-analysis on vegan diets and multiple health outcomes found evidence that conformity to exclusive vegetarian and vegan diets was associated with reduced overall cancer risk^23^. Different study designs, subjects' characteristics, lack of controlling for several confounders and different sample sizes across studies might explain the differences between our findings and those of earlier studies. Overall, further research using the PDI, hPDI, and uPDI or other holistic approaches might help resolve these inconsistencies.

The biological rationale for the possible association of plant-based diet on BC risk might be as follow: higher intake of healthy plant-products including fruits, legumes, vegetables and whole grains is accompanied with higher intake of fiber, micronutrients including vitamins C, E, folate, carotenoids, trace minerals (selenium, zinc, copper, and manganese), some non-nutrient such as phytoestrogens, phenolic acids, and lignans^57^ and carotenoids which are known to affect cellular differentiation and apoptosis^58^. Dietary folate, by its role in DNA methylation, affects gene expression of critical tumor suppressors and proto-oncogenes^59^. Dietary fiber plays important protective role against cancer through estrogen modulatory effects^60,61^. In addition, dietary fiber was found to be associated with a reduced cancer risk through removing damaged cells from the digestive dilute bile acids, and thereby it can reduce cell proliferation and the likelihood of mutations^62^. Reducing *N*-nitroso compounds, enhancing immunity, and producing various anti-inflammatory cytokines involved in the initiation and progression of mammary cell growth were also attributed to the anti-carcinogenic effects of dietary fiber^63^. Vitamins C, E, and trace minerals such as selenium, zinc, copper, and manganese are key constituents for antioxidant enzymes^29^. Essential non-nutrients, such as phytoestrogens, phenolic acids, and lignans with a modulatory effect on hormonal pathways through antioxidant, antiproliferative, antiangiogenic, and apoptotic properties play important protective roles against cancer^64^. On the other hand, animal products, mostly red and processed meat, are rich sources of heme iron, which through the production of genotoxic free radicals, NOCs, or through lipid peroxidation might have carcinogenic effects^65^. Moreover, elevated IGF-1 levels resulting from higher intake of high-protein animal foods can also play a role in this regard. This factor has been linked with both obesity and the development of various types of cancer. The mitogenic, anti-apoptotic, and angiogenic properties of IGF-1, particularly in mammary cell lines, have previously been confirmed^66,67^.

Several advantages of this project include adequate sample size, adjustment for a wide range of potential confounders in the analysis, reduced chance of altered usual dietary intakes by the enrollment of newly diagnosed BC cases, being the first investigation in the Middle East region and using energy-adjusted amounts of all food groups for constructing plant-based diet indices. Given these strengths, some potential limitations also need to be pointed out. The present study was carried out in a case–control design, which is prone to recall and selection biases and would not allow us to infer causality. However, this study was a population-based study in which controls were chosen from the healthy population in the community, which can in turn reduce selection bias. As in any epidemiological study, the application of FFQ might result in participants’ misclassification in terms of dietary intakes. Given the potential for existing unmeasured or unknown confounders, despite comprehensive adjustment for many potential confounders, the possibility of residual confounding cannot be ignored. We implemented sample-based scoring of plant-based diets to evaluate conformity to different types of plant-based diets; however, due to the lack of clear thresholds for absolute levels of plant and animal foods intake concerning chronic conditions, this field needs further investigation. Finally, we did not collect information on BC stage in the current study.

In conclusion, findings of the present study indicated that greater adherence to PDI and hPDI were inversely associated with risk of BC, whereas uPDI was associated with an increased risk. These findings support current recommendations for increasing the intake of healthy plant-based foods in dietary guidelines. Further studies are needed to confirm our findings, especially those with a prospective design.
